# The Rates of Breastfeeding in Baby-Friendly Hospitals in Greece: A Nationwide Survey

**DOI:** 10.3390/children9121792

**Published:** 2022-11-22

**Authors:** Eftychia Liakou, Evangelos Christou, Nicoletta Iacovidou, Abraham Pouliakis, Rozeta Sokou, Chrysa Petropoulou, Paraskevi Volaki, Anastasia Triantafyllou, Matina Zantiotou, Dionisios Vrachnis, Theodora Boutsikou, Zoi Iliodromiti

**Affiliations:** 1 Neonatal Department, Medical School, National and Kapodistrian University of Athens, Aretaieio Hospital, 115 28 Athens, Greece; 2 Second Department of Pathology, Medical School, National and Kapodistrian University of Athens, “Attikon” University Hospital, 124 61 Athens, Greece; 3 Second Department of Obstetrics and Gynecology, Medical School, National and Kapodistrian University of Athens, Aretaieio Hospital, 115 28 Athens, Greece

**Keywords:** infant, exclusively breastfeeding, breastfeeding, baby friendly hospital, WHO indicators

## Abstract

Background: Exclusive breastfeeding (EBF) remains the cornerstone of infant nutrition for the first six months of life, presenting multiple short and long term benefits. The purpose of this study is the demonstration of EBF rates of infants born in baby-friendly hospitals (BFH) and the factors that positively influence EBF. Methods: The study was conducted in all four of the BFH that exist in Greece, between 2020 and 2022. The study sample consisted of 1200 mothers, taken from the 7101 that delivered at those hospitals during the time of the study. A questionnaire was used that included questions to evaluate the infant’s nutrition after birth, after exiting the maternity hospital and during the 2nd, 4th and 6th month of age. The WHO guidelines on EBF and breastfeeding (BF), as well as the “Infant and Young Child Feeding” indicators, were used. Results: The EBF rate within 1 h after birth was 71.3%, which gradually declined to 21.2% in the 6th month. The respective rate of BF was 94.5% and declined to 66.1%. The logistic regression revealed that attending antenatal breastfeeding courses, vaginal delivery, full-term pregnancies and the mothers’ advanced education level constitute independent positive prognostic factors for increased EBF rates. Conclusion: The results of the first national study on BFH are presented. Despite the improvement of EBF rates in Greece, compared to the latest available data from 2018, reinforcement of EBF promotion measures is required in order to approach the WHO’s targets by 2025.

## 1. Introduction

All major health organizations, including the World Health Organization (WHO), the United Nations International Children’s Emergency Fund (UNICEF) and the American Academy of Pediatrics, unanimously recommend exclusive breastfeeding (EBF) for the first six months of life, as breast milk’s nutritional content and bioactivity is considered optimal to promote healthy development. Despite the undisputed benefits of breast milk nutrition, only 23 countries have achieved EBF rates of approximately 60% worldwide, while the overall percentage of EBF younger than 6 months old is particularly low, accounting for 40% in total [1].

The protection against infections has been well evidenced during lactation, especially during the first six months of life, as breastfeeding (BF) exhibits lower morbidity rates against: acute otitis media, by 23%; upper respiratory tract infections, by 63%; gastroenteritis; and urinary tract infections. Moreover, BF is associated with a lower risk of type I diabetes, mellitus, obesity, malignancy during adulthood, and rates of sudden infant death syndrome [2,3,4].

In developing countries, infants that consume cow’s milk-protein-based formulas demonstrate a 14-fold increase in mortality rate due to gastrointestinal infections and a 4-fold increase in mortality rate due to respiratory tract infections, in comparison to BF infants. Although the beneficial effect of BF is especially evident in developing countries, only 37% of infants under 6 months old receive [5].

According to studies, BF harbors more diverse intestine microbiota, which is usually dominated by Bacillales, Lactobacillales and Clostridiales species in early life, in comparison to formula-fed infants. The intestine microbiome of breastfed infants that are vaginally delivered (VD) constitute the gold standard of a healthy microflora. Compared to neonates delivered with a cesarian section (CS), they demonstrate a unique bacterial colonization pattern and microbial diversity that is intrinsic to healthy immune maturation and function [6].

The American Academy of Pediatrics currently supports the use of breast milk, or donor human milk in cases where the mother’s own milk is not sufficient, for all premature infants hospitalized in NICUs [7].

The Baby Friendly Hospital Initiative, launched by WHO and UNICEF in 1991, aims to create a healthcare environment in which breast-feeding is the normal. This is accomplished by enabling mothers to make an informed choice and by supporting early initiation of breast-feeding. Maternity hospitals that are recognized as baby-friendly hospitals (BFH) in Greece are: the “Aretaieio” University Hospital, the General-Maternity District Hospital “Elena Venizelou”, the “Attikon” General University Hospital and the General Hospital of “Preveza”.

In 2017, the Child Health Institute of Greece, in collaboration with the National Center of Public Health, conducted a National Survey in order to evaluate the frequency and main determining factors of EBF, as well as to assess the progression of BF in Greece [8].

According to the results of the aforementioned survey, EBF was determined to account for 16.8%, until the fifth month of age; while the same rate was <1% by the end of the sixth month. Moreover, during the sixth month, the BF rate among participants was found to be 45%.

The purpose of this study is the demonstration of breastfed infant’s rates in BFH in Greece during five chronological milestones of the infant’s life: (a) the first day; (b) at the discharge from the maternity hospital; (c) during the 2nd month; (d) during the 4th month; (e) during the 6th month. This study is intended to give an overview of data collection in terms of the basic determining factors and markers regarding BF, and aims to collect information in regard to the BFH modus operandi.

## 2. Materials and Methods

### 2.1. Design

The study was conducted in the four BFH in Greece, namely the “Aretaieio” University Hospital, the “Attikon” General University Hospital, the General-Maternity District Hospital “Elena Venizelou” and the General Hospital of “Preveza”. The study was approved by the respective Hospitals Scientific Committees (219/18-06-20, 479/24-08-20, 19608/03-09-20, 62/29-09-20) and took place between 1 October 2020 and 30 January 2022. All participating mothers gave written consent prior to their participation in this study. The study was conducted in accordance with the STROBE statement [9]. 

### 2.2. Sample

Two trained researchers undertook the task of conducting an interview with the participating mothers, after obtaining their informed written consent. The study group consisted of 1200 mothers out of the 7101 that potentially fit the inclusion criteria during the time of the study. Prior to the official start of the study, a total of ten mothers from each hospital participated in a brief pilot assessment in order to evaluate the validity of the structured questionnaire and were, thereupon, excluded from the study. During the course of the study, one mother was excluded due to her infant’s death. The exclusion criteria were in cases of multiple pregnancies, maternal or fetal morbidity during pregnancy, maternal underlying conditions that potentially affect lactation and neonatal morbidities, and the mother’s age during pregnancy being <15 years of age.

### 2.3. Data Collection

The study was conducted using a structured questionnaire, designed by the research team. The participants were women who gave birth at the aforementioned BFH during the study period, after providing an informed written consent form. The questions concerned five different time periods: during the infant’s delivery at the maternity hospital, before exiting the maternity hospital and during the 2nd, 4th and 6th month of age. The completion of the questionnaire that refer to the last 3 consecutive time periods was conducted via phone interviews. 

The questionnaire is structured with closed-ended dichotomous questions and the respondents have to choose among predefined answers (YES/NO) and multiple choice questions that aim to optimize the stratification of answers and ensure the participants’ anonymity. It consists of three categories: the first of which refers to the obstetric history (22 questions), the second is about the perinatal history (8 questions) and the third assesses the mother’s medical history (11 questions). (Supplementary File S1).

In accordance with the WHO’s guidelines [10], EBF refers to the percentage of infants who receive only breast milk and no other form of foods or liquids, with the exception of oral rehydration solutions, drops, and syrups (vitamins, minerals, medicines). BF refers to the percentage of infants who receive breast milk with or without any other type of food or drink, including breast milk substitutes (non-human milk and formula). 

All BFH that are recognized by the Ministry of Health in Greece are obliged to conform to rather strict criteria, imposed by the WHO, regarding their mode of operation [11]. The “Attikon” General University Hospital and the General-Maternity District Hospital “Elena Venizelou” were officially recognized in 2011; the “Aretaieio” University Hospital in 2016; while the General Hospital of “Preveza” was recognized in 2020.

### 2.4. Statistical Analysis

The data were recorded in Microsoft Excel spreadsheets (Microsoft Corporation, Redmond, Washington, DC, USA), in rows that correspond to pairs of maternal and neonatal data. Statistical analysis was performed via the SAS for Windows 9.4 software platform (SAS Institute Inc., Cary, NC, USA). Baby weight (the sole arithmetic variable) was expressed as median and quartile 1 (Q1), quartile 3 (Q3) range and the other variables (categorical) were expressed as frequencies and the relevant percentages. Comparisons of percentages were performed via the z-test. Multivariate analysis was performed via logistic regression and the results are expressed as the relevant Odds Ratio (OR), the 95% Confidence Interval (CI) and the *p*-value. The significance level (*p*-value) was set to 0.05 and all tests were two sided. As the number of women that delivered infants in the aforementioned hospitals (i.e., all BFH in Greece) during the study period was 7101 in total, the sample size of 1200 mothers is considered representative of the population (i.e., all women that delivered in baby-friendly hospitals in Greece) as the error margin is 3%. The sampling was randomized for each participating hospital and was based on a selection of one in every five women. Due to the random sampling, we expect that the population is represented in terms of the mothers’ age and socioeconomic status, as well as in other characteristics.

## 3. Results

### 3.1. Sample Characteristics

The majority of the participants were mothers belonging to the 31–40 year old age group (57.2%), whilst the 15–18 year old group had the fewest participants (1.6%). Amongst the participants, 53.7% had given birth at least once before. Overall, 61% delivered by CS, while 39% delivered vaginally (either normally or via vacuum-assisted VD). Table 1 presents the socio-demographic characteristics of participants. Additionally, Table 2 shows the reasons the mothers refrained from attending antenatal BF courses. It is noted that only 18.7% of the total participants attended such courses during the current pregnancy. On the contrary, 36.2% of them had attended antenatal courses during a previous pregnancy.

### 3.2. BF Rates

**Figure 1:**
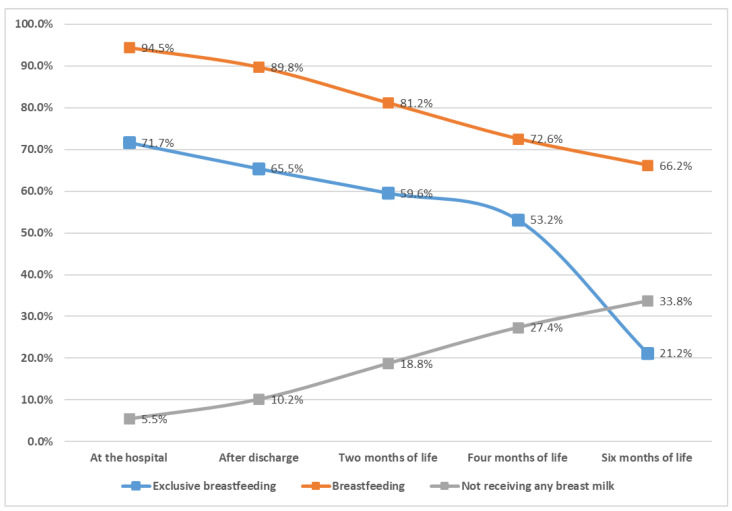
Breastfeeding indicators by infants’ age in the national breastfeeding prevelance study at baby friendly hospital in Greece.

Figure 1 presents the respective rates of EBF, BF and neonates who did not receive any breast milk during the five pre-defined time periods chosen in this study. A gradual decline in the EBF rates from birth (71.3%) until the 6th month of age (21.2%) is observed. In addition, the analysis demonstrates a progressive decrease (lesser from EBF) in the percentages of BF from birth until the 6th month of age, where it accounts for 66.1%, respectively.

### 3.3. Factors Influencing EBF and BF Rates

The majority of participants delivered by CS in VD, as already stated. Table 3 presents a comparison between the rates of EBF and BF according to mode of delivery, during the respective time periods of the study. Women that delivered vaginally were more likely to engage in EBF, compared to those that delivered by CS, in all time periods of the study. Mothers who delivered vaginally displayed statistically significant increased BF rates in all time periods of the study, in comparison to mothers that delivered by CS.

Additionally, the relationship between early initiation of BF and EBF and rates of BF was studied by analyzing the respective rates of cases in which BF was initiated within the first hour after birth (skin-to-skin contact), within the first day of birth but after the first hour, or after the first day of birth. The results are analytically described in Table 4. According to the study results, neonates that received skin-to-skin contact and initiated BF within the first hour after birth demonstrated increased EBF rates in all time periods of the study, in comparison to neonates that initiated BF after the first day of birth. Furthermore, neonates that initiated BF within the first hour after birth exhibited increased EBF rates, in comparison to neonates that initiated BF within the first day of birth but after the first hour (with the exception of the exit from the maternity hospital, *p* = 0.051). Additionally, a statistically significant greater number of neonates received BF than those who initiated BF within the first hour after birth and those that initiated BF after the first day of life at the 4th and 6th month. There is no statistically significant difference in the rates of BF between the neonates that initiated BF within the first hour of birth and those that initiated BF the first day but after the first hour. 

The current study also examined the relationship between gestational age at delivery and the probability of EBF and BF. According to our study results, 90.9% of the neonates were born at term; 8.1% were born late pre-term (born between 34 and 36 full weeks of pregnancy); and 1% were born pre-term (born at less than 34 weeks of pregnancy). No statistically significant association between pre-term and late pre-term neonates regarding EBF and BF emerged. On the other hand, fewer pre-term neonates received EBF compared to term neonates at the hospital, after the hospital exit and the 2nd month. Moreover, less pre-term neonates received BF compared to term neonates during their stay at the maternity hospital and during the 6th month of age. No significant differences between the groups were noted during the other time periods of the study. The results are illustrated in Table 5. In Table 6, it is evident that neonates whose mothers attended antenatal breastfeeding courses demonstrated statistically significant increased EBF and BF rates in all time periods of the study.

### 3.4. Logistic Regression with Reference to the Factors That Influence EBF:

The logistic regression regarding factors that have a positive impact on EBF is shown analytically in Table 7. It appears that attending antenatal BF courses accounts for the two-fold higher odds of the mothers succeeding in EBF, in comparison to mothers that had not attended such courses. On the contrary, mothers that delivered by CS demonstrate decreased odds, by 50%, of achieving EBF, compared to mothers that delivered vaginally. It was also noticed that although pre-term and late pre-term neonates did not display a statistically significant discrepancy concerning EBF rates, term neonates achieved higher rates of EBF in all time periods of the study in comparison to pre-term neonates. However, the aforementioned rates tend to converge towards the 6th month of age, at which point the odds decline. The initiation of BF within the 1st hour after birth plays a crucial role in the strengthening of BF in the first months of neonate’s life, as the results from the logistic regression show us. Finally, women who have received a higher education level committed to EBF to a greater extent than women that have received a basic education level, yet they displayed higher odds compared to women that have obtained a high school diploma and those that had basic knowledge about EBF.

## 4. Discussion

Despite the fact that the European Union holds the lowest BF rates globally [12], at present, BF has been proven to offer indisputable health benefits and long term positive effects, especially in cases of EB during the first 6 months of age [13]. To date, our study is the first in Greece to investigate the EBF and BF rates among infants born exclusively in BFH, until the 6th month of age. The study is conducted nationwide, and it involves a large number of participants. In comparison with the latest available data published in Greece regarding BF, that derive from a National Survey conducted by the Child Health Institute of Greece in 2018 [8], whose main objective was to assess the prevalence of EBF in Greece and to identify its main contributing factors, our study illustrates a positive trend in EBF rates in all time periods of the study. The main difference between the present study and the aforementioned studies is that our study sample consisted of mothers that delivered exclusively in BFH, whilst the former included participants that delivered indiscriminately in all public hospitals, regardless of its baby-friendly accreditation.

### 4.1. EBF Rates during the First 6 Months

It is already acclaimed that the WHO has set a global target to increase the rate of EBF during the first six months, to at least 50% by 2025 [14]. Although a relative increase in the possibility of EBF has been observed in BFH versus the rates of EBF in neonates that were delivered in all public hospitals (21,2% vs 0.8%) [8], the current rate is still rather low and, thus, distant from the proclaimed target.

Similarly, the prevalence of EBF is sub-optimal in Greece, compared to most European countries, even in BFH where skin-to-skin contact between the newborn and mother immediately after birth is common practice. The documented rate of EBF after birth in Greece, at 71.5%, is significantly lower than the respective rate of other European countries, notably, Croatia (93%), Denmark (97%), Germany (86%), the Netherlands (89%), Sweden (80%) and Switzerland (95%). On the contrary, Ireland (46%) and Norway (68%) display even poorer rates of EBF [15].

Additionally, a remarkable decrease was observed in EBF rates during the first six months. Specifically, EBF rates declined from 71.7% at birth to 65.6% after exiting the maternity hospital. Relatively high EBF rates reported in the initial period gradually declined to 59.6% during the 2nd month, followed by precipitous drops to 53.2% and 21.2% during the 4th and 6th month, respectively, despite the ESPGHAN [16] and other pediatric societies’ recommendations for EBF during the first six months. The substantial decrease in EBF rates observed in our study is in accordance with international rates, documented both in the USA, where EB during the 6th month is achieved in only 24.9% of infants [17], and in the European Region, where the respective rate is an estimated, almost identical, 25% of infants being exclusively breastfed for the first six months [18]. The main reason for the low EBF rates is the inadequate implementation of the Ten Steps to successful BF [19], as well as the primary health care system’s failure to provide and ensure a supportive environment, for both the mother and the newborn, for the promotion and establishment of BF. To make matters worse, certain healthcare professionals’ lack of interest, empathy and knowledge concerning the undeniable advantages of BF could also attribute to the problem. In Greece, the major problem remains the lack of midwives and health visitors specialized in BF who support breastfeeding mothers in the post-partum period. Therefore, the mothers usually cannot face the difficulties and desist from the BF. 

### 4.2. Which Are the Key Points for the Promotion of EBF?

Our study established a correlation between the mode of delivery and the EBF rates. Neonates delivered via CS, rather than by VD, achieved lower EBF rates in all time periods of the study [20,21]. It is reported that the post-operative care routines after CS have a detrimental effect on BF outcomes, as they delay the skin-to-skin contact between the mother and her newborn within the first hour after birth and they cause the mother physical and psychological stress, which consequently leads to a reduction in breast milk production and often results in the replacement of EBF with supplementary formula feeding.

We confirmed that the early initiation of BF within one hour of birth is ultimately associated with higher EBF rates, compared to both when the initiation of BF occurs within the first day of birth but not within the first hour of birth, and when the initiation occurs after the first day of birth, a claim that is also stated in similar studies [22,23]. According to the WHO, the early initiation of BF within the first hour of birth constitutes a core indicator of infant and young child feeding [24], as, in addition to its well documented positive effect on EBF until the 6th month, it ensures the neonate’s exposure to the mother’s microbiota, which offers immunostimulant properties, results in a more optimal thermoregulation, and improves maternal-infant bonding [25]. The present study did not document differences between the rates of BF in the neonates that initiated BF within the first hour of birth and those that initiated BF after the first hour within the first day of life. This may be a random finding in our study and requires more studies focused on BF, rather than EBF, in the future in order to investigate the validity of this result. Hence, the implementation of specific policies by BFH, in terms of supporting the early initiation of BF within the first hour of birth and avoiding supplementary feeding for supposed medical reasons, play a pivotal role towards this direction.

Another remarkable observation was that neonates whose mothers attended antenatal breastfeeding courses were almost twice as likely to achieve EBF in all time periods of the study, in comparison to neonates whose mothers refrained from attending. In a number of instances, the participants suggested that the COVID-19 pandemic posed a barrier to participating in such courses, either due to the postponement of the class or their inability to attend, proving that the application of methods such as the conduction of online courses failed to provide an effective solution. On the other hand, even though attending antenatal or perinatal courses is certainly beneficial, there are numerous studies highlighting that the initiative of creating a supportive environment within the maternity hospital premises towards promoting EBF plays a dominant role. However, it is worth mentioning that the participation in such courses is significant as the longer the duration, the higher the EBF rates [26]. Although the antenatal classes reduce the rate of CS, improve the rates of BF and create an advantageous environment for the BF, it is known that in the postpartum period, they are not enough [27]. The importance of a supportive net of the family from the maternity hospitals after the birth and the social services of the state with the participation of pediatricians, midwives and health visitors in the post-partum period remains the milestone [28]. In Greece, the antenatal classes of BF organized from the BFH weekly in small groups (<10 participants). The duration lasts two months. The objective of the classes is the physiology of BF, the anatomy of breasts, the indications-contraindications of BF, the nutrition of the mother, and the psychological support of family. The right to participation has been ensured to the candidate father. The classes contain a theoretical and practical part. during COVID-19, the classes occurred online. Although in recent years we have observed a progression in this field, we need much more. 

Our study ascertained that mothers who have received a higher education level present a higher probability to commit to EBF than women that have received basic education level, which agrees with the results from other studies [29,30]. The lack of awareness concerning the benefits of BF, the significantly limited access to public antenatal healthcare services attributed to their accompanying poor socioeconomic status and the inadequate antenatal care they receive have been reported to contribute to the low level of EBF. 

## 5. Limitations

A major limitation in our study is that not all regions of the country are equally represented as three out of four maternity hospitals, from which our sample derives, are located in the capital and only one is located in a province (Preveza). Nevertheless, this was inevitable because those are the only certified BFH in Greece. We succeeded in maintaining high retention and participation rates throughout the duration of the study, as only one participant from the original sample discontinued due to her infant’s demise, at two months. Additionally, it is probable that a selection bias in the selection of the original sample has occurred on account of the inherently emotional nature of BF and the potential hesitation of a mother to admit that they failed to successfully breastfeed. Therefore, the two assigned researchers that were responsible for completing the questionnaire used “nutrition of neonates and infants during the first six months” as a general interview title. Moreover, sample representativeness was taken into consideration prior to the initial selection of participants in order to create a more diverse and inclusive sample in terms of the mothers’ age and socioeconomic status. In addition, communication barriers due to language were resolved with the aid of the hospital’s translation services. Finally, the study was conducted during the pandemic, which restricted the recruitment of even more participants because of the applied restrictive measures.

## 6. Conclusions

The current study offers a thorough and detailed update on nutrition characteristics of infants during the first six months of age in Greece. Additionally, it provides crucial insight into the positive impact of BFH and the factors that effectively influence EBF.

Although the prevalence of EBF in Greece has recently shown a positive trend, in the period from birth until the 6th month, compared to the latest available data from 2018, for infants born in BFH who were exposed to policies that support and promote EBF, the global target the WHO has set for 2025 remains distant and demands collective effort and determination to be approached. An effort to reinforce public healthcare policies, raising awareness amongst healthcare professionals to encourage and familiarize future mothers towards the undisputable benefits of EBF and the establishment of safe and supportive conditions that would protect maternity in our country and would facilitate mothers to breastfeed are required.

## Figures and Tables

**Table 1:** Characteristics of mothers and infants in the study.

Characteristic	Metric, *n* (%)
Maternal Age (in years)	
15–18	20 (1.6%)
18–30	422 (35.3%)
31–40	685 (57.3%)
41–48	70 (5.8%)
Family status	
Single	119 (9.9%)
Married	1081 (90.1%)
Maternal educational attainment	
Primary School	106 (8.8%)
High school	568 (47.4%)
University	524 (43.8%)
Maternal employment status during pregnancy	
Yes	645 (53.7%)
No	555 (46.3%)
First pregnancy	
Yes	560 (46.6%)
No	640 (53.4%)
Mode of delivery	
Cesarean section	732 (61%)
Vaginal delivery	468 (39%)
Previous experience of breastfeeding	
Yes	608 (50.6%)
No	592 (49.4)
Baby weight (g) median [Q1–Q3 range]	3140 [2880–3430]

**Table 2:** Characteristics about the antenatal courses of breastfeeding.

Question	*n* (%)
Mothers attended antenatal classes during the current pregnancy	
Yes	224 (18.7%)
No	976 (81.3%)
Why didn’t mothers attend antenatal classes of breastfeeding?	
COVID-19	366 (37.5%)
Insufficient knowledge	256 (26.2%)
Not interested	28 (2.8%)
No free time	210 (21.5%)
Language difficulties	19 (1.9%)
Not applicable	32 (3.3%)
Maternal health issue	56 (5.8)
Other reason	9 (1%)
Mothers attended antenatal classes during a previous pregnancy	234 (36.2%)
Mothers knew before their admission that the hospital they delivered is certified as a “Baby Friendly Hospital”	828 (69.4%)

**Table 3:** The relationship between the mode of the delivery and the rates of the exclusive breastfeeding, the breastfeeding and the infants who did not receive any breast milk by infants’ age.

Delivery Type.	Exclusive Breastfeeding	Breastfeeding	Not Receiving Any Breast Milk	Total	*p* *	*p* **
At the hospital						
VD	383 (81.84%)	451 (96.3%)	17 (3.7%)	468	<0.0001	0.0267
CS	477 (65.25%)	682 (93.3%)	49 (6.7%)	731		
Total	860 (71.73%)	1133 (94.5%)	66 (5.5%)	1199		
After hospital exit						
VD	357 (76.28%)	433 (92.5%)	35 (7.5%)	468	<0.0001	0.0140
CS	428 (58.55%)	644 (88.1%)	87 (11.9%)	731		
Total	785 (65.47%)	1077 (89.8%)	122 (10.2%)	1199		
Two months after birth						
VD	326 (69.81%)	408 (87.4%)	59 (12.6%)	467	<0.0001	<0.0001
CS	388 (53.08%)	565 (77.3%)	166 (22.7%)	731		
Total	714 (59.6%)	973 (81.2%)	225 (18.8%)	1198		
Four months after birth						
VD	297 (63.73%)	369 (79.2%)	97 (20.8%)	466	<0.0001	<0.0001
CS	340 (46.51%)	500 (68.4%)	231 (31.6%)	731		
Total	637 (53.22%)	232 (72.6%)	328 (27.4%)	1197		
Six months after birth						
VD	115 (24.68%)	348 (74.7%)	118 (25.3%)	466	0.0235	<0.0001
CS	139 (19.02%)	445 (60.7%)	286 (39.3%)	731		
Total	254 (21.22%)	793 (66.2%)	404 (33.8%)	1197		

Note: *: *p* value refers to the vaginal delivery vs cesarean section for the exclusive breastfeeding. **: *p* value refers to the vaginal delivery vs cesarean section for the breastfeeding between VD and CS.

**Table 4:** The relationship between the time of the first breastfeeding and the rates of the exclusive breastfeeding, the breastfeeding and the infants who did not receive any breast milk by infants’ age.

Time of First Breastfeed	Exclusive Breastfeeding	Breastfeeding	Not Receiving Any Breast Milk	Total	*p* *	*p* **	*p* ^†^	*p* ^‡^
At the hospital								
1st hour	648 (78.64%)	790 (95.9%)	34 (4.1%)	824 (69.8%)	0.0110	0.3417	<0.0001	0.3770
After the 1st hour and within 24 h	181 (70.7%)	249 (97.2%)	7 (2.8%)	256 (21.7%)				
After 24 h	31 (31%)	94 (94%)	6 (6%)	100 (8.5%)				
Total	860 (72.9%)	273 (23.1%)	47 (4%)	1180 (100%)				
After hospital exit								
1st hour	589 (71.5%)	748 (90.7%)	76 (9.3%)	824 (69.8%)	0.0517	0.5920	<0.0001	0.2751
After the 1st hour and within 24 h	166 (64.8%)	235 (91.8%)	21 (8.2%)	256 (21.7%)				
After 24 h	30 (30%)	94 (94%)	6 (6%)	100 (8.5%)				
Total	785 (66.5%)	1077 (91.2%)	103 (8.8%)	1180 (100%)				
Two months after birth								
1st hour	533 (64.7%)	683 (83%)	140 (17%)	823 (69.8%)	0.0403	0.7115	<0.0001	0.4544
After the 1st hour and within 24 h	147 (57.4%)	210 (82%)	46 (18%)	256 (21.7%)				
After 24 h	34 (34%)	80 (80%)	20 (20%)	100 (8.5%)				
Total	714 (60.56%)	973 (82.5%)	206 (17.5%)	1179 (100%)				
Four months after birth								
1st hour	492 (59.85%)	627 (76.2%)	195 (23.8%)	822 (69.8%)	0.0001	0.1549	<0.0001	0.0001
After the 1st hour and within 24 h	116 (45.31%)	184 (71.8%)	72 (28.2%)	256 (21.7%)				
After 24 h	29 (29%)	58 (58%)	42 (42%)	100 (8.5%)				
Total	637 (54%)	869 (73.7%)	309 (26.3%)	1178 (100%)				
Six months after birth								
1st hour	200 (24.3%)	580 (70.5%)	242 (29.5%)	822 (69.8%)	0.0303	0.0533	0.0009	<0.0001
After the 1st hour and within 24 h	45 (17.6%)	164 (64.1%)	92 (35.9%)	256 (21.7%)				
After 24 h	9 (9%)	49 (49%)	51 (51%)	100 (8.5%)				
Total	254 (21.6%)	793 (67.3%)	385 (32.7%)	1178 (100%)				

Note: * *p* value refers to the first breastfeeding within 1st hour vs within 24 h (but not first hour) for the exclusive breastfeeding. ** *p* value refers to the first breastfeeding within 1st hour vs within 24 h (but not first hour) for the breastfeeding. ^†^ *p* value refers to the first breastfeeding within 1st hour vs after 24 h for the exclusive breastfeeding. ^‡^ *p* value refers to the first breastfeeding within 1st hour vs after 24 h for the breastfeeding.

**Table 5:** The relationship between the gestational age of the newborn and the rates of the exclusive breastfeeding, the breastfeeding and the infants who did not receive any breast milk by infants’ age.

Gestational Age	Exclusive Breastfeeding	Breastfeeding	Not Receiving Any Breast Milk	Total	*p* *	*p* **	*p* ^†^	*p* ^‡^
At the hospital								
<34 weeks	1 (10%)	8 (80%)	2 (20%)	10	0.1605	0.1661	<0.0001	0.0413
34–36 + 6 weeks	37 (37.8%)	91 (92.8%)	7 (7.2%)	98				
≥37 weeks	822 (75.3%)	1034 (94.7%)	57 (5.3%)	1091				
Total	860 (71.7%)	1133 (94.5%)	66 (5.5%)	1199				
After hospital exit								
<34 weeks	1 (10%)	10 (100%)	0 (0%)	10	0.1780	0.2913	0.0003	0.2844
34–36 + 6 weeks	36 (36.7%)	88 (89.8%)	10 (10.2%)	98				
≥37 weeks	748 (68.5%)	979 (89.7%)	112 (10.3%)	1091				
Total	785 (65.4%)	1077 (89.8%)	122 (10.2%)	1199				
Two months after birth								
<34 weeks	2 (20%)	8 (80%)	2 (20%)	10	0.5994	0.5649	0.0158	0.8632
34–36 + 6 weeks	33 (33.7%)	70 (71.4%)	28 (28.6%)	98				
≥37 weeks	679 (62.3%)	895 (82.1%)	195 (17.9%)	1090				
Total	714 (59.6%)	973 (81.2%)	225 (18.8%)	1198				
Four months after birth								
<34 weeks	2 (20%)	5 (50%)	5 (50%)	10	0.9441	0.6278	0.0505	0.0843
34–36 + 6 weeks	26 (26.5%)	57 (58.2%)	41 (41.8%)	98				
≥37 weeks	609 (55.92%)	807 (74.1%)	282 (25.9%)	1089				
Total	637 (53.22%)	869 (72.6%)	328 (27.4%)	1197				
Six months after birth								
<34 weeks	2 (20%)	3 (30%)	7 (70%)	10	0.3356	0.2078	0.8533	0.0109
34–36 + 6 weeks	6 (6.1%)	50 (51%)	48 (49%)	98				
≥37 weeks	246 (22.6%)	740 (67.9%)	349 (32.1%)	1089				
Total	254 (21.2%)	793 (66.2%)	404 (33.8%)	1197				

Note: * *p* value refers to the newborns gestational age <34 weeks’ vs 34–36 + 6 weeks for the exclusive Breastfeeding. ** *p* value refers to the newborns gestational age <34 weeks’ vs 34–36 + 6 weeks for the breastfeeding. ^†^ *p* value refers to the newborns gestational age <34 weeks’ vs ≥37 weeks for the exclusive breastfeeding. ^‡^ *p* value refers to the newborns gestational age <34 weeks’ and ≥37 weeks for the breastfeeding.

**Table 6:** The relationship between the attendance of antenatal breastfeeding classes and the rates of the exclusive breastfeeding, the breastfeeding and the infants who did not receive any breast milk by infants’ age.

Attended Classes	Exclusive Breastfeeding	Breastfeeding	Not Receiving Any Breast Milk	Total	*p* *	*p* **
At the hospital						
Yes	354 (83.9%)	411 (97.4%)	11 (2.6%)	422	<0.0001	0.0011
No	506 (65.1%)	722 (92.9%)	55 (7.1%)	777		
Total	860 (71.7%)	1133 (94.5%)	66 (5.5%)	1199		
After hospital exit						
Yes	339 (80.3%)	405 (96%)	17 (4%)	422	<0.0001	<0.0001
No	446 (57.4%)	670 (86.5%)	105 (13.5%)	777		
Total	785 (65.5%)	1077 (89.8%)	122 (10.2%)	1199		
Two months after birth						
Yes	317 (75.1%)	385 (92.2%)	37 (8.8%)	422	<0.0001	0.0008
No	397 (51.2%)	588 (75.8%)	188 (24.2%)	776		
Total	714 (59.6%)	973 (81.2%)	225 (18.8%)	1198		
Four months after birth						
Yes	291 (69.1%)	359 (85.3%)	62 (14.7%)	421	<0.0001	0.0477
No	346 (44.6%)	510 (65.7%)	266 (34.3%)	776		
Total	637 (53.2%)	869 (72.6%)	328 (27.4%)	1197		
Six months after birth						
Yes	122 (29%)	339 (80.5%)	82 (19.5%)	421	<0.0001	0.0010
No	132 (17%)	454 (58.5%)	322 (41.5%)	776		
Total	254 (21.2%)	793 (66.3%)	404 (33.7%)	1197		

Note: * *p* value refers to the mothers who attended antenatal breastfeeding classes vs those who did not attend. ** *p* value refers to the mothers who attended antenatal breastfeeding classes vs those who did not attend.

**Table 7:** Multivariate analysis about the indicators of the exclusive breastfeeding by infants’ age.

	At the Hospital		After Hospital Exit		Two Months after Birth		Four Months after Birth		Six Months After Birth	
Effect	OR (95% CI)	*p*	OR (95% CI)	*p*	OR (95% CI)	*p*	OR (95% CI)	*p*	OR (95% CI)	*p*
Attended antenatal courses (yes vs no)	1.93 (1.39–2.69)	<0.0001	2.06 (1.53–2.79)	<0.0001	1.93 (1.46–2.55)	<0.0001	1.95 (1.5–2.54)	<0.0001	1.71 (1.34–2.18)	<0.0001
Delivery method (CS vs VD)	0.54 (0.4–0.74)	0.0002	0.53 (0.39–0.7)	<0.0001	0.53 (0.41–0.7)	<0.0001	0.63 (0.48–0.81)	0.0003	0.73 (0.58–0.93)	0.0107
Gestational age (34–36 + 6 w vs <34 w)	3.5 (0.94–13.11)	0.7728	1.67 (0.46–6.02)	0.8066	0.85 (0.24–3.06)	0.2083	1 (0.27–3.71)	0.3639	1.25 (0.32–4.97)	0.65
Gestational age (37w vs <34w)	9.84 (2.72–35.54)	<0.0001	3.34 (0.97–11.57)	0.0058	1.85 (0.54–6.37)	0.0421	1.99 (0.56–7.07)	0.0475	2.24 (0.59–8.53)	0.0567
Education level (high school vs school)	2.77 (1.73–4.42)	0.0133	4.02 (2.57–6.28)	0.0001	4.08 (2.6–6.39)	0.0011	2.81 (1.78–4.43)	0.0372	2.64 (1.65–4.23)	0.0646
Education level (university vs school)	3.53 (2.14–5.84)	<0.0001	5.3 (3.29–8.53)	<0.0001	6.53 (4.06–10.52)	<0.0001	4.36 (2.7–7.02)	<0.0001	4.11 (2.52–6.7)	<0.0001
Family status (married vs single)	1.45 (0.93–2.26)	0.1035	1.68 (1.11–2.54)	0.0146	2.02 (1.34–3.03)	0.0007	2.33 (1.54–3.5)	<0.0001	1.82 (1.2–2.77)	0.0049
1st breast feeding (after 1 h vs. within 1 h)	0.65 (0.48–0.89)	0.0079	0.81 (0.6–1.09)	0.1628	0.91 (0.68–1.21)	0.5015	0.69 (0.53–0.91)	0.0078	0.75 (0.58–0.99)	0.0392

## Data Availability

All the data are included in the study.

## Supplementary Materials

The following supporting information can be downloaded at: https://www.mdpi.com/article/10.3390/children9121792/s1, Supplementary File S1: The questionnaire of the survey.

## Abbreviations

ΕΒF	Exclusive breastfeeding
WHO	World Health Organization
BF	Breastfeeding
BFH	Baby Friendly Hospital
CS	Cesarean section
VD	Vaginal Delivery
