# Supplementary material for: The Rates of Breastfeeding in Baby-Friendly Hospitals in Greece: A Nationwide Survey
Source: Children (Basel). 2022 Nov 22;9(12):1792. doi: 10.3390/children9121792 (PMC9777116; doi:10.3390/children9121792)
Supplement: Supplementary file 1 [file children-09-01792-s001.zip › children-2022679-supplementary.pdf]

SURVEY QUESTIONNAIRE

- The questions that are marked as follows: ☐ refer to single-answer questions.
- The questions that are marked as follows: ◯ refer to multiple-answer questions.

A. Obstetric History

1. In which hospital did you receive your antenatal care and give birth?

☐ “Aretaeio” University Hospital
☐ General Maternal Hospital “Elena Venizelou”
☐ “Attikon” University Hospital
☐ Preveza General Hospital

2. Which is the gestational age of your current pregnancy (in weeks)?

☐ < 34w
☐ $34\text{-}36^{+6}$ w
☐ ≥ 37w

3. Your current pregnancy is:

☐ Singleton
☐ Twin
☐ Other..

4. Is it your first pregnancy?

☐ Yes
☐ No

5. Which is the mode of delivery?

☐ Normal vaginal delivery
☐ Vacuum-assisted vaginal delivery
☐ Caesarean section

6. How many other children do you have?

- [ ] 0
- [ ] 1
- [ ] 2
- [ ] 3
- [ ] Other..

(In case your answer is 0 please refrain from answering the following question).

7. Have you breastfed any of your other children?

- [ ] Yes
- [ ] No

8. If so, for how long?

- [ ] <1 month
- [ ] 1 – 3 months
- [ ] 3 - 6 months
- [ ] 6 - 12 months
- [ ] >12 months

If not, what was the reason?...

9. What kind of analgesia were you administered during labor?

- [ ] An epidural block
- [ ] Pethidine
- [ ] GeneralAnesthesia
- [ ] Nothing
- [ ] Other..

10. What was your newborn's weight of birth? BW:...

☐ < 2500g

☐ 2500-3999g

☐ ≥4000g

11. What was the newborn's weight at discharge from the hospital? BW at discharge:.....................................

12. Was it necessary to be separated from the newborn immediately after birth due to medical reasons?

☐ Yes

☐ No

13. If so, what was the reason?

◯ Prematurity

◯ Neonatal Illness (perinatal asphyxia)

◯ Neonatal congenital anomalies

◯ Other……………………………………………………….

(If not, proceed to the next question)

14. When did you place your newborn on your breast?

☐ During the first hour after birth

☐ During the first day after birth

☐ After the first day after birth

Which of the following describes better the way of feeding your baby?
Mark an "X" in the appropriate box.

	**During your stay at the maternity hospital**	**After your discharge from the hospital**	**2 months**	**4 months**	**6 months**
**Exclusive breastfeeding**					
**Formula Feeding**					
**Breastfeeding and Supplementing Formula Feeding**					

15. Which were the reasons your newborn was given supplementing or exclusive breast milk substitutes during your stay at the hospital?

○ Maternal inability to breastfeed

○ Neonatal inability to breastfeed

○ Maternal medical reasons

○ Neonatal medical reasons

○ Other...

16. Have you encountered any of the following problems while breastfeeding?

○ Mastitis

○ Nipple lesions

○ Sore nipples

○ Breast milk inadequacy

○ Medication / Maternal

○ medical conditions

○ None of the above

○

All of the above

Other...

17. Have you used lactation supplements to increase your breast milk supply?

☐ Yes

☐ No

18. Who helped you during the lactation period?

◯ Neonatologists/Pediatricians

◯ Obstetricians/Gynecologist

◯ s

◯ Maternity nurses/Nurses

◯ Family/Friends

◯ On my own

Other...

19. How would you characterize the help you received concerning breastfeeding during your stay at the hospital, provided you received such help?
Mark an "X" in the box for the answer that is closest to your opinion.

**Poor 1**	**Inadequate 2**	**Medioc re 3**	**Adequat e4**	**Very adequat e 5**

20. After being discharged for the maternity hospital did you seek help for breastfeeding at home?

☐ Yes

☐ No

21. If so, who did you turn to for help?

◯ A health professional
◯ A telephone support line
◯ Internet

◯ Family/Friends

◯ Other..

22. If so, in which manner?

- Home visitation
- Using telephone communication
- Using digital communication/mail

23. Has it been easy for you to find special nursing rooms in public places in order to breastfeed your baby undisturbed during a stroll with your baby?

- Yes
- No

B. PERINATAL HISTORY

25. Have you attended antenatal classes of breastfeeding during the current pregnancy?

- Yes
- No

26. If not, what was the reason?

- Lack of time
- Covid-19
- Other...

27. If so, which class did you attend?

- “Aretaeio” University Hospital
- General Maternal Hospital “Elena Venizelou”
- “Attikon” University Hospital
- Preveza General Hospital
- Other...

28. Have you attended antenatal classes of breastfeeding during a previous pregnancy?

- Yes
- No

29. Were you aware of the fact that the maternity hospital in which you were admitted and delivered your baby is a certified “Baby friendly hospital” before your admittance?

- Yes
- No

30. Who informed you about the existence of the “Baby friendly hospitals” that exist in Greece?

◯ A health professional
◯ Family/Friends
◯ A TV/radio program
◯ Internet

31. Were you informed about the following during your stay at the **maternity hospital?:**

◯ Skin to skin contact
◯ Rooming in
◯ The benefit of breastfeeding
◯ All the above
◯ None of the above
◯ Other..

32. In case you were informed about any of the above, who did so and how?

◯ A health professional
◯ Family/Friends
◯ Other..

Γ. MEDICAL HISTORY

33. How old are you (years)?

☐ <18
☐ 18-30
☐ 31-40
☐ 41-48
☐ >48

34. What is your marital status?

☐ Married
☐ Cohabitation
☐ Agreement
Single

- [ ] Single parent family
- [ ] Divorced / Separated
- [ ] Living together

35. What is your highest level of education?

Primary School

- [ ] Secondary School
- [ ] Further education public institution
- [ ] Bachelor's University degree
- [ ] Postgraduate degree (Master's/ Doctorate)
- [ ] Other..

36. Are you currently employed?

- [ ] Yes
- [ ] No

37. If so, what is your employment status?

- [ ] Employed in the private domain
- [ ] Employed in the public domain
- [ ] Self-employed
- [ ] Other...

38. Have you taken a maternity leave during your current pregnancy?

- [ ] Yes
- [ ] No
- [ ] Dismissal
- [ ] Work from home
- [ ] Other ....

39. Have you taken a child-raising leave?

- ☐ Yes
- ☐ No
- ☐ Dismissal
- ☐ Work from home
- ☐ Other ....

40. Is the father of the child employed?

- ☐ Yes
- ☐ No

41. If so, what is his employment status?

- ☐ Employed in the private domain
- ☐ Employed in the public domain
- ☐ Self-employed
- ☐ Other...

42. What is your combined annual income (euros)?

- ☐ ≤ 10.000
- ☐ 10.001-20.000
- ☐ 20.001-30.000
- ☐ 30.001-40.000
- ☐ > 40.001

43. What is your nationality?

- ☐ Greek
- ☐ Albanian
- ☐ Russian
- ☐ Bulgarian
- ☐ Romanian
- ☐

Moldavian

Arabic

- [ ] East Asian
- [ ] Other...
